# Temporal fractals in movies and mind

**DOI:** 10.1186/s41235-018-0091-x

**Published:** 2018-03-14

**Authors:** James E. Cutting, Jordan E. DeLong, Kaitlin L. Brunick

**Affiliations:** 1 Department of Psychology, Uris Hall, Cornell University, 109 Tower Road, Ithaca, NY 14853-7601 USA; 2 Research Narrative, LLC, Los Angeles, CA USA; 3 ZERO TO THREE, Washington, DC, USA

## Abstract

Fractal patterns are seemingly everywhere. They can be analyzed through Fourier and power analyses, and other methods. Cutting, DeLong, and Nothelfer (2010) analyzed as time-series data the fluctuations of shot durations in 150 popular movies released over 70 years. They found that these patterns had become increasingly fractal-like and concluded that they might be linked to those found in the results of psychological tasks involving attention. To explore this possibility further, we began by analyzing the shot patterns of almost twice as many movies released over a century. The increasing fractal-like nature of shot patterns is affirmed, as determined by both a slope measure and a long-range dependence measure, neither of which is sensitive to the vector lengths of their inputs within the ranges explored here. But the main reason for increased long-range dependence is related to, but not caused by, the increasing vector length of the shot-series samples. It appears that, in generating increasingly fractal-like patterns, filmmakers have systematically explored dimensions that are important for holding our attention—shot durations, scene durations, motion, and sound amplitude—and have crafted fluctuations in them like those of our endogenous attention patterns. Other dimensions—luminance, clutter, and shot scale—are important to film style but their variations seem not to be important to holding viewers’ moment-to-moment attention and have not changed in their fractional dimension over time.

## Significance

Psychologists are very good at studying the instant or an instantaneous slice out of a longer episode. Yet we often have very little to say about how information, or how a mental activity, is distributed over 1 h or more. Popular movies offer an opportunity to investigate such distributed information and mental activity and the linkage between them. Movies have: (1) shots that vary in duration which are separated by cuts that dictate eye movements; (2) scenes that vary in duration which control event structure and attention to the narrative; and varying (3) degrees of motion and (4) sound amplitude that also affect attention. In an investigation of 295 movies released from 1915 to 2015, we find that the film-length patterns of these four dimensions of movies have converged over the last 50–80 years on temporal fractal patterns (1/*f*) and we find others that have not. These differences suggest that the fractal patterns are, in some sense, intentional on the part of filmmakers. Moreover, these results can be mapped onto their statements about the goals of their craft – to synchronize viewers’ attention with the rhythms of the movies. These 1/*f* patterns also mimic the fluctuations of attention shown in cognitive tasks (Gilden, 2001, 2009), suggesting that movie viewers’ attention patterns are not that different from those found in the laboratory.

## Background

The photoplay … will thus become more than any other art the domain of the psychologist who analyzes the workings of the mind.

Münsterberg (1915, p. 31)

The photoplay obeys the laws of the mind rather than those of the external world.

Münsterberg (1916, p. 97)

Hugo Münsterberg was an applied psychologist who, late in his life, became infatuated with the then-new art form of photoplays; we now call them movies. He exhorted psychologists to study and to make movies for the purposes of exploring the human mind. It appears that no professional psychologist followed his suggestion, perhaps largely because there were few tools and little technology available to undertake such a scientific study. But time has passed and our statistical and computational means have vastly improved. Now, after a lapse of a century, an increasing number of psychologists are interested in the psychology and cognitive science of movies (see, for example, Kaufman & Simonton, 2014; Shimamura, 2013; see also Hochberg & Brooks, 1996). Münsterberg was certainly correct to suggest that movies could be used to study the mind (see, for example, Bezdek et al., 2015; Hasson, Malach, & Heeger, 2009; Levin & Baker, 2017; Magliano & Zacks, 2011) but likely wrong to separate the laws of the mind from those of the external world.

In this context, Cutting, DeLong, and Nothelfer (2010) reported a striking finding. In an analysis of the patterns of shot durations across the lengths of 150 different popular movies released from 1935 to 2005, they found a trend in fractal-like temporal patterns. That trend had two parts. From 1935 to about 1960, there was considerable variation across movies and little apparent relation of fractal dimension to those movies but, over the period from about 1960 to 2005, shot-duration fluctuations began to approach a fractal-like pattern. The theoretical account for the division of movies into these two groups is a standard one in film studies: Hollywood movies from the silent era to about 1960 were produced top-down under the studio contract system and those thereafter were increasingly produced by more independent groups of individuals assembled ad hoc for each movie (see Bordwell, 2006; Bordwell, Staiger, & Thompson, 1985).

The Cutting et al. results were striking because Gilden (2001, 2009; Gilden, Thornton, & Mallon, 1995, Thornton & Gilden 2005; see also Pressing & Jolley-Rogers, 1997) had earlier reported that a fractal patterning was found in choice reaction times for cognitive tasks. Could there be a functional connection between the structure of movies, which require exogenous shifts of attention, and psychological laboratory tasks, which require endogenous emissions of attention? One purpose of this article is to suggest further that there may be.

Complicating the search for a connection is the problem that fractals are everywhere, in both time series and in visible arrays. Large numbers of entities in nature and culture seem to follow these self-similar patterns (DeLong, 2015; Gilden, 2009; Mandelbrot, 1983; Newman, 2005; West, 2017) – the measurement of coastlines, the fluctuations in stock markets, the variations in the height of tides, the branching of trees, the florets in Romanesco broccoli, and the patterns in music, speech, steps, breaths, heartbeats, and so forth. Perhaps we should assume that fractality (Stadnitski, 2012a) is the null hypothesis when considering naturally or socially occurring, complex temporal or spatial structure. If this were the case, the ubiquity of fractals also makes it more difficult to determine a functional linkage between any pair of them. Let us outline the outstanding issues, our path to discovery, and then explore the nature of fractals in time-series data.

### Continuing issues about movies and fractals

Again, Cutting et al. (2010) reported that fluctuations of shot durations in movies have become increasing fractal-like and that this might be related to attention. Three issues remain unsettled. First, the increase in measured fractal dimension might be contaminated by the length of the data vector, as we discuss below. Second, fractal vectors imply distal correlations in the data, but the use of a power spectrum analysis may not be the most advantageous to demonstrate such long-range dependence, which we discuss in Studies 1 and 2. And third, there is currently only a weak linkage between fractal dimensions in movies, which exogenously demand attention, and the fractal dimensions of data in cognitive tasks, signifying fluctuations in endogenous attention. We attempt to address this in our concluding discussion.

To us, the most intriguing aspect of the results of Cutting et al. (2010) is that, insofar as we knew at the time, we had documented the only increase in fractal-like structure over time (but see Wijnants, Bosman, Hasselman, Cox, & Van Orden, 2009). To be sure, we had no firm account of why this might be so, but it seemed likely to be enabled by the increased availability of film footage that could be cut into a film (giving editors more choices) and, over the last 30 years, by the increased use of digital, non-destructive editing techniques. The latter afford greater speed and precision and a greater ease in the reworking of visual ideas. Of course, the underlying assumption is that, somehow, film editors and likely other filmmakers tacitly have in mind the ideal of a fractal-like pattern of shot durations for the whole film.

The article by Cutting et al. (2010) has been reasonably widely cited, particularly in the press, and it garnered wide attention on the Internet. Unfortunately, in their interpretation of our article, bloggers often made two errors. First, they linked the results to the alleged shrinking of viewers’ attention spans, for which there is no evidence. And second, they thought that an increasing fractal-like dimension improved the quality of the movies, garnering higher profits, which Cutting et al. had assessed and for which they also found no evidence.^1^ The paper also attracted attention within the community of cinemetrics scholars, those who use quantitative methods to measure certain aspects of movies.^2^

The most important critique evolved out of several cinemetric discussions. Salt (2010) suggested that faster editing (shorter shots), particularly in action movies, might by itself create increasing slopes. Cutting and Salt went back and forth on this and other issues, and Cutting (2014c) reanalyzed the data from 160 movies from 1935 to 2010 and found reliable effects of both release year (again a quadratic effect) and number of shots. Meanwhile, DeLong (2015) performed many analyses on the movie sample used by Cutting et al. (2010), replicating the original findings and going beyond them. However, DeLong’s analyses reinforced Salt’s (2010) speculation. Several measures of fractal dimension seemed sensitive to sample size (in other words, to the number of shots in the movie). With these suggestive but inconclusive leads, it seemed time to revisit the idea of shot fluctuations in popular movies as inherently moving towards a fractal pattern.

## Path of narration and discovery

In this article, we present something of a twisted tale. We first give a short background concerning fractal (fractional) analysis of time series vectors, with an emphasis on those related to the shot patterns of popular movies. We then move on to six empirical studies.

In Study 1, we replicate the results of Cutting et al. (2010) while nearly doubling the number of popular movies investigated and by extending the time frame of the corpus of movies. In particular, we find that the fluctuations of shot durations in movies after 1960 have increasingly approached a temporal fractal pattern (1/*f* ^1^; see, for example Mandelbrot, 1999). However, we also find two important constraints. First, our major result – that after 1960 the increased slope, α, of the shot-duration fluctuations fit by our model 1/*f* ^α^ − is also strongly correlated with the number of shots in the movies. Second, investigating the broader literature, we discovered that the major aspect of our results – long-range dependence – may not be best measured by our model.

In Study 2, we substitute for our power-spectrum model a different measure of linear vector complexity – the exact local Whittle estimator. This is an algorithm used for parameter estimation in autoregression analyses and is regarded as a good estimate of the fractional (fractal-like) nature of a vector. Using the Whittle estimator, we replicate two aspects of our results from Study 1. First, we find an increase in the magnitude of Whittle estimates over time and, second, the Whittle estimate is also correlated with the number of shots per movie. The issue raised, then, is: Are both measures contaminated by the length of the vector analyzed?

Study 3 simulates vectors of different lengths and different fractal-like values and, for the relevant range of fractality, we find little general increase in either slopes or Whittle estimates with increase in vector length within our domain of study. Similarly, Study 4 doubles the lengths of the shot vectors in all movies and finds no general increase in either slope or Whittle estimates compared to the original data. Thus, although the length of the shot vector is correlated with the fractal-like results of Studies 1 and 2, it is not a cause underlying those results of increasing vector complexity over time. In addition, Study 3 showed that the variability in the slope estimates is considerably greater than that for the Whittle estimates, thus suggesting that the latter provides a more consistent measure of fractal-like structure in a vector.

Study 5 investigated three additional fluctuations in movies that, like shot duration, show a confluence over time towards a fractal dimension – the duration patterns of scenes, the motion patterns across shots, and the sound amplitude patterns. Over release years, we find striking linear increases in the Whittle estimates for scene durations and motion, and a decrease for sound amplitude, with all three of these measures converging towards a true fractal.

Study 6 investigated fluctuations in movies that show no changes over time in fractality – the patterns of luminance, clutter, and shot scale. That is, results show no convergence toward a true fractal over release years. Finally, we link these results to statements by filmmakers and to psychological responses. Next, we need to elucidate the nature and structure of fractal vectors.

## Fractals, time series, and colored noise

A fractal (or a fractionally dimensioned object; Mandelbrot, 1983) contains a pattern that repeats at many different scales, from small to large and vice versa. Thus, fractals are called self-similar. Here we focus on temporal patterns in time-series data. When analysis is done on a temporal fractal, the power of each Fourier component increases in proportion to its wavelength – the inverse of its frequency (or 1/*f*). Thus, patterns in larger component sine waves are scaled-up versions of those of smaller component sine waves; they are enlarged equally in both wavelength and the square of the amplitude (that is, power). Phase is not relevant in this context.

**Fig. 1:**
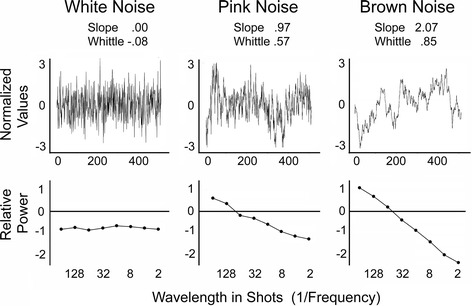
Three types of temporal noise. The *top panels* show 512-element samples of white (or random) noise, pink (or fractal) noise, and brown (or Brownian) noise. *Slope values* refer to the exponent (alpha) in the expression 1/*f* ^α^ and Whittle values refer to the exact local Whittle estimator of long-range dependency in the data (Shimotsu & Phillips, 2005). See the text for explanations of both. The *bottom panels* show the power spectra for each patch of noise for wavelengths (traveling windows along the time-series vector) between 2^8^ to 2^1^ shots

Consider the three waveforms shown in upper panels of Fig. 1. By tradition these are called noises. The different noise arrays were generated by an algorithm given in Little, McSharry, Roberts, Costello, and Moroz (2007), the output values were then normalized (mean = 0, standard deviation = 1), and then their fractal property remeasured. The upper left panel shows an array of random numbers called white noise, the upper middle panel shows numbers in a fractal pattern called pink noise, and the upper right panel shows brown noise, akin to one-dimensional Brownian motion.

Together, these are called colored noises. These seem to be strange terms and the origins of some of these names may appear obscure. To be sure, we already noted the origin of the “color” brown from Brownian motion, but the others are less clear. White is from analogy to white light, which has roughly equal energy at all visible frequencies, and pink stems from the fact that light with a fractal distribution appears pink, with strongest components at the long-wavelength (red) end of the chromatic spectrum.

**Fig. 2:**
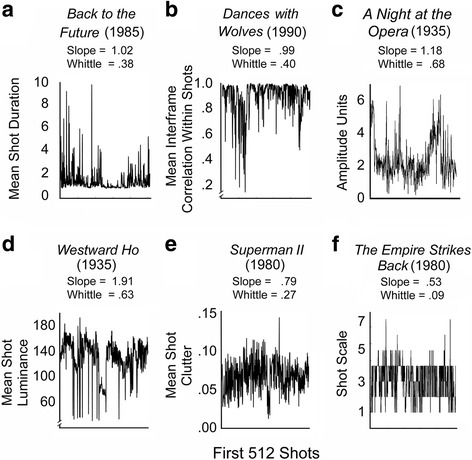
Waveforms for six dimensions of movies – **a** shot duration, **b** motion, **c** sound amplitude, **d** luminance, **e** clutter, and **f** shot scale – taken from the first 512 shots in six movies. The first and waveforms like it are the focus of Studies 1, 2, and 4; the latter five and waveforms like them from other movies are discussed in Studies 5 and 6. Slope = the value of alpha in 1/*f* ^α^; Whittle = a fractional estimate of vector complexity. Both are discussed in the text

When plotted on log-log coordinates of power against frequency (the inverse of wavelength), white, pink, and brown noises have different slopes: white ≅ 0.0, pink (or fractal) ≅ 1.0, and brown ≅ 2.0. Functions with these approximate slopes are as shown in the lower panels of Fig. 1. In white noise, every value is independent of the one that precedes it; in brown noise (also called a random walk or a drunkard’s walk), every value is randomly generated around the previous value. Pink noise is “in between.” We will discuss these as noises with different slopes, where the slopes (ideally 0, 1, and 2, but varying smoothly in between) are given by exponent alpha in the power-spectrum expression 1/*f* ^α^. Notice that two of the slopes at the bottom of Fig. 1 are negative rather than positive but, by convention and since the exponent in the expression is in the denominator, this reverses the sign (and direction of slope). Figure 1 also reports the values of the exact local Whittle estimators of these noises, a measure we discuss in detail in Study 2. The types of “noises” that we will consider, however, look quite different than those in Fig. 1. Nonetheless, these can be measured in the same way once the data are normalized. Some of these are shown in panels of Fig. 2.

The top panels refer to dimensions investigated in Studies 1 and 5. Figure 2a shows the series of shot durations for the first 512 shots of *Back to the Future* (Zemeckis, 1985) which, for the complete vector of 1327 shots, has a fractal slope near 1.0. Figure 2b shows the relative amount of motion in each of the first 512 of 2468 shots in *Dances with Wolves* (Kostner, 1990), which also has a slope near 1.0. Motion here is measured as the mean correlation between the luminance values of all pixels in successive frames where all of those frames occur within a given shot. Thus, 1.0 is perfect stillness and 0.2 is a low mean correlation of frames (a lot of motion) within a shot. Values, of course, can be as low as −1.0, but few movies have any shots with interframe correlations < 0. Figure 2c shows the sound amplitude profile across the length of *A Night at the Opera* (Wood, 1935) of the first 512 of 1281 values, each representing 100 frames (a 4.17-s slice) of the movie. The pattern is measured in arbitrary amplitude units. Here the slope is quite steep, > 1.0.

The bottom panels refer to dimensions discussed in Study 6. Figure 2d shows the mean within-shot luminance for the first 512 of 548 shots in *Westward Ho* (Bradbury, 1935). Here the slope is very steep, near 2.0 and close to brown noise. Figure 2e shows the mean clutter in the first 512 of 1887 shots in *Superman II* (Lester, 1980), where clutter is measured as the proportion of edge pixels in the image that remain after each frame is passed through Laplacian of Gaussian filter (see Henderson, Chanceaux, & Smith, 2009; Rosenholtz, Li, & Nakano, 2007). And finally, Fig. 2f shows the shot scale profile for the first 512 of 1782 shots in *Star Wars: Episode 5 – The Empire Strikes Back* (Kershner, 1980). Notice the discrete steps of 1 to 7 in the shot scales. Its slope is about halfway between white and pink noise.

## Study 1: Shot-duration fluctuations, sample size, and long-range dependence

### Methods

#### Assembling a larger sample of movies

Members of our lab have studied many quantitative aspects of movies, incrementally increasing the sample size as we have progressed. Much of this is discussed and reviewed in Cutting (2016a). Cutting et al. (2010) analyzed the shot-duration patterns of 150 English-language, feature-length, popular movies – ten each for 15 years evenly divisible by five (e.g. 1935, 1940, …, 2000, 2005). We sampled across genres and from among the most popular of these release years. Subsequently, we expanded that sample to include ten similarly chosen movies from 1915, 1920, 1925, and 1930, and ten from 2010 and 2015.

For other purposes, we had replaced ten of these movies that were longer than 2.5 h. These are thought to have different narrative properties than those under that limit (Thompson, 1999). The alternates were ten with more standard durations from the same genres and release years. Nevertheless, here we have included both the originals and the ten alternates. We also added two from Cutting, DeLong, and Brunick (2011). This aggregation, so far, yields 222 movies released over a century, 1915 to 2015. A listing of 210 of these is given in Cutting (2016b), ten more can be found in the supplementary material to Cutting et al. (2010), and two in Cutting, DeLong and Brunick (2011).

To these we added 75 separate feature-length movies made for children and explored by Brunick (2014). Three per year, these were released between 1985 and 2008 and were the highest grossing G-rated theater or direct-to-DVD releases. Two of these films overlapped with the previous aggregate, yielding a total of 73 different movies. The children’s movies have remarkably similar shot-pattern characteristics to the movies made for adolescents and adults for the same period (Brunick & Cutting: Pace and appearance in movies made for children and adults, in preparation), which provides a list of those movies.

In sum, we now had a grand total of 295 English-language, feature-length movies, almost twice that of Cutting et al. (2010). Many analyses below, however, are done on 263 movies, and some on 180, 48, and 24. Thus, the statistical power for determining effects – where α = 0.05, and *d* = 0.80 – is 0.99+, 0.99+, 0.99+, 0.77, and 0.46, respectively, for samples of 295, 263, 180, 48, and 24 movies. The median effect size reported in this article is *d* = 0.72.

#### Measuring time-series power spectra

With one exception, we followed the methods used by Cutting et al. (2010). We created a vector consisting of the linear sequence of shot durations in each movie. All movies in this sample had between 188 and 3235 shots, as determined in previous research. The movies were between 49 and 204 min in duration. The Academy of Motion Picture Arts and Sciences defines a feature-length film as one lasting at least 40 min. However, except for the silent movies (1915–1925, mean duration = 81 min) and the children’s movies (mean duration = 89 min) in this sample, the mean duration of the other feature movies is quite constant at about 110 min from 1930 to 2015. Again, the values in these shot-duration arrays were then normalized for each movie.

The next step entailed Fourier analysis; this was accomplished by fitting phase-shifted sine waves to successive and successively larger segments (windows) of the shot vector. The lengths of these shot windows were powers of two – 2 shots, 4 shots, 8, 16, 32, 64, 128, and up to 256 shots. We fit travelling windows of each size along the length of the shot vector. That is, for example, for segment lengths of eight shots we fit the normalized durations of shots 1 to 8, then 2 to 9, then 3 to 10, then 4 to 11, and so forth through *n*-7 to *n*, where *n* is the number of shots in the movie. We then averaged these separate fits, calculating mean power.

**Fig. 3:**
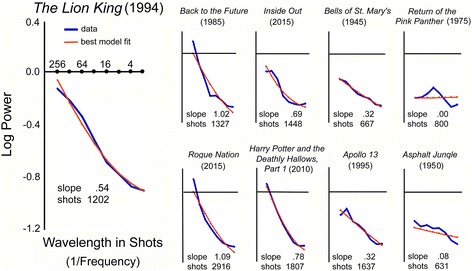
Power spectra for the data (in *blue*) of and model fits (in *red*) to the shot-duration fluctuations of nine movies. These reflect a power analysis on the normalized shot vectors for each movie. Notice three trends: steeper-sloped movies tend to be more recent, more recent movies tend to have more shots, and model fits tend to be better for movies with more shots. These parallel trends form the focus of Studies 1–4

Cutting et al. (2010) had extended their analyses to 2^m^ shots, where *m* is the largest power of 2 that is less than the *n* shots in each movie. They then fit these data with a hybrid model that measured both white noise (or random noise, which has a flat spectrum, and is referred to as 1/*f* ^0^) and colored noise. Their assumption, and that of Gilden (2001; see also Wijnants et al., 2009), was that all such signals have a background of random (white) noise and that a fractal-like pattern should be estimated as emerging in the context of that background. Importantly, for the colored-noise part of the model, we varied alpha (α) in 1/*f* ^α^ until the simultaneous combination of colored noise and white noise best fit the data. All obtained values of alpha for these movies were in the range of 0.0–1.54, with a mean of 0.55 and a standard deviation of 0.25. Some of these fits are shown in Fig. 3 here and others were shown in Fig. 3 of Cutting et al. (2010).

If the size of the shot sample inherently increases the exponent alpha, as suggested in previous discussions and research (Cutting, 2014c; DeLong, 2015; Salt, 2010), this might be because the increase in the number of samples in each window and that the averages over the larger number of samples reduces statistical variability, yielding smoother and more reliable functions. To explore this possibility, we truncated the power analysis after travelling windows of 2^8^ (or 256) shots but analyzed the shot vector out to *n*, its last shot. Thus, the larger the *n* the more the averages should smooth the results. In addition, we analyzed only those movies with at least 512 shots. This latter criterion reduced the sample to 263 movies.

### Results

#### Slopes and individual movies

Figure 3 shows the data and model fits for nine movies. *The Lion King* (Allers & Minkoff, 1994), a movie of 1202 shots, provides a framework for the display of the others. By convention and as in Fig. 3, the traveling window sizes (wavelengths, or 1/frequencies) appear on the abscissa in descending order (256 to 2 shots). These are plotted against the relative log power values on the ordinate. The data are shown by a thicker blue line. The model fit (combining white and colored noise) is shown by a thinner red line. Notice that several model fits are slightly curved, as they should be with a log-scaled mixture of white noise (a flat function) and colored noise (a sloped function). The influence of the white noise would diminish with greater wavelengths and greater power. The slope of the colored noise fit (α in 1/*f* ^α^) for *The Lion King* is 0.54, about halfway between a true fractal (1/*f* ^1^) and white noise (1/*f* ^0^).

Given this backdrop, a full range of data and model fits from eight other movies are also shown in Fig. 3. Notice that the slopes (the values of alpha in 1/*f* ^α^) are near 1.0 for the leftmost pair of movies (*Back to the Future*, Zemeckis, 1985, and *Mission: Impossible – Rogue Nation*, McQuarrie, 2015), near 0.67 for the next two movies (*Inside Out*, Docter & Del Carmen, 2015, and *Harry Potter and the Deathly Hallows, Part 1*, Yates, 2010), near 0.33 for the third pair (*Bells of St. Mary’s*, McCarey, 1945, and *Apollo 13*, Howard, 1995), and near zero for the rightmost pair (*Return of the Pink Panther*, Edwards, 1975, and *Asphalt Jungle*, Huston, 1955). Notice, too, that the more recent movies are generally to the left and that they also generally have more shots.

Thus, the results for these nine movies set up the pattern for both effects – that more recent movies have a steeper slope, in line with the results of Cutting et al. (2010), but they also have more shots. And a third effect is that across all movies the increase in the number of shots is correlated with the improvement of the hybrid model fits (adjusted *R*^2^ = 0.05, *t*(261) = − 3.81, *p* = 0.0002, *d* = 0.47). Mean root-mean-squared deviations for movies with about 500 shots is about 0.20, whereas that for those with about 2000 shots is about 0.10. Notice that the fit for *Return of the Pink Panther* is particularly poor.

#### Expectations and the patterns of slopes across movies

**Fig. 4:**
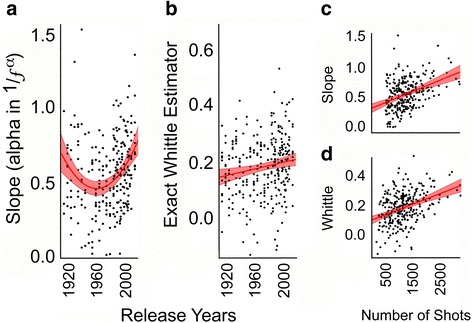
Results of Studies 1 and 2 plotting values of fractal measurements per movie against release years and against number of shots per movie. Shown are four *scatterplot* results for movies released between 1915 and 2015. **a** Alpha values (slopes) of 263 movies as a quadratic function of release year (Study 1). Only the right half of that function fits the data well. **b** The exact Whittle estimate values for 295 movies as a linear function of release year (Study 2). **c** The slopes as a function of the number of shots in 263 movies (Study 1). **d** The Whittle estimates for 295 movies as a function of the number of shots (Study 2). *Colored areas* are 95% confidence intervals on the regressed fits

Cutting et al. (2010) reported that the pattern of slopes among the earlier movies (from 1935 to about 1960) was relatively flat and varied and that the pattern for the later movies (about 1960 to 2005) increased over time with less variation. Cutting et al. also reported that the linear increase across the whole set, 1935 to 2005, was also reliable, but not as compelling. With the movies added to the beginning of the release year distribution (1915, 1920, 1925, and 1930) and to its end (2010 and 2015) it was difficult to know what we should predict. More critically, however, the addition of the children’s movies increased the density of movies between 1985 and 2008, roughly the time frame of the sharpest increase in slope, which was the central and emphasized finding of Cutting et al. The results are shown in Fig. 4a.

Based on previous results, we looked for both linear and quadratic trends. To be clear, the data are quite noisy, which is the main reason for waiting eight years to update Cutting et al. (2010) until we could explore many films over a longer period of time. An increasing linear trend was modest (adjusted *R*^2^ = 0.017, *t*(261) = 2.34, *p* = 0.02, *d* = 0.30), but the quadratic trend shown in the figure was more robust (adjusted *R*^2^ = 0.087, *t*(260) = 4.60, *p* < 0.0001, *d* = 0.57).

Again, the quadratic trend bottoms out at about 1960, a result that would appear to reinforce the division of popular movies into those of the Hollywood Studio era and those that came later. However, the left-hand side of the trend has little statistical support. Although the apparent decline in slopes from 1915 to 1955 looks impressive, it is not by itself reliable (adjusted *R*^2^ = 0.045, *t*(60) = − 1.68, *p* = 0.098). Thus, the quadratic function fails the two-lines test (Simonsohn, 2017) – dividing the distribution between falling and rising segments, and testing for the significance of both linear trends. Nonetheless, our interest had originally been focused on the period from 1960 onwards.

Importantly for the argument presented in Cutting et al. (2010), the linear trend of the subsample from 1960 to 2015 was also quite strong (adjusted *R*^2^ = 0.11, *t*(200) = 5.10, *p* < 0.0001, *d* = 0.72). Thus far, then, our evidence extends the results of Cutting et al. (2010).

#### Slopes and shot-sample size

In exploration of the effects of shot number (sample size), Fig. 4c shows the scatterplot of the same slope values against the number of shots in each movie. The regression trend, with its 95% confidence interval, is quite strong (adjusted *R*^2^ = 0.15, *t*(261) = 6.78, *p* < 0.0001, *d* = 0.84), replicating Cutting (2014c). As one can see, the mean slope (alpha value) for movies with only about 500 shots is near 0.4, but for those with 3000 shots is near 1.0. Clearly, as was seen in the individual movie data of Fig. 3, both release year and shot number are contenders in accounting for the data.

Using two predictors of slope, the quadratic regression values from the release-year data of each movie and the linear regression values for the number of shots, we find that shot number is a stronger predictor (*t*(260) = 4.84 *p* < 0.0001, *d* = 0.60) than is release year (*t*(260) = 2.49, *p* = 0.014, *d* = 0.30). Indeed, in stepwise regression, we find that entering the number of shots first accounts for 15% of the variance and the addition of the quadratic values adds only 2%, whereas entering the quadratic values first yields 9% of the variance, but the addition of shots adds another 8%. Thus, it is clear that the number of shots, not release year, is the more potent cause of the increase in slope.

Moreover, and again, since the pattern in the post-1960 movies was most critical to the conclusions of Cutting et al. (2010), we could simply assess the linear effects of release year and shot number on derived slope in those movies from 1965 to 2015. Together these account for 24% of the variance in the data, but the effect of shot number is again substantial (*t*(189) = 5.89, *p* < 0.0001, *d* = 0.86), whereas that of release year is not (*t*(189) = 1.89, *p* = 0.06). Clearly, the evolution towards a 1/*f* ^1^, or fractal, structure in the shot patterns of movies is reflected in more shots per movie in these data than in release years.

Why do longer shot vectors garner higher slope values? Again, one reason might be a smoothing of the data through the averaging of more samples. As can be seen in Fig. 3, the fits of the hybrid model to the data seem to get better as the number of shots increases (right to left). On the other hand, one might have assumed that the mean slope estimates would remain roughly the same across movies with different numbers of shots, but with decreased variance (not increased slope) as the number of shots per movie increased. This possibility is one rationale behind the simulations in Study 3.

### Long- and short-range dependence

An important issue emerges from the broader literature in the context of these data and analyses. This concerns long-range dependence, also called – and seemingly in a deliberate ploy to confuse psychologists – “memory.” The idea comes from hydrology and originally concerned the cadence of the build-up of runoff from rainstorms throughout a watershed as the water approached a dam on a large river (Hurst, Black, & Simaika, 1965). Over the subsequent decades this idea was then applied to many time-domain self-similar processes, even brain states (Tagliazucchi et al., 2013).

To be concrete, the implication of this idea to the results of Study 1 and those of Cutting et al. (2010) is as follows: they claimed that there are long-range relationships among the shot durations. But that claim may be suspect. In particular, the relatively high power in the long-wavelength results of *The Lion King*, seen at the left-hand side of Fig. 3, suggests that, among others, there are correlations among the shot durations at lags of 256 shots that are due to long-range processes underlying the data. As it turns out, however, this need not be the case. Short-range dependence (local correlations) can lead to effects that look like long-range processes are at work (Karagiannis, Faloutsos, & Riedl, 2002; Wagenmakers, Farrell & Ratcliff, 2005).

This is a known problem, an active research area, and has been addressed in many different venues (see DeLong, 2015) – for example, Karagiannis et al. (2002) in telecommunications research and Wagenmakers et al. (2005; Farrell, Wagenmakers, & Ratcliff, 2006) in response to Gilden (2001) and his study of reaction times. Both sets of authors offered solutions. Wagenmakers et al. suggested testing the difference in autoregressive (AR) model results [ARFIMA(1,*d*,1) - ARMA(1,1)] on each data vector. The first model has a component (*d* for dimension, not effect size) that could measure long-range dependence, but the second model does not. However, Gilden (2009) questioned this approach on grounds of model flexibility and the overfitting of data.

On the other hand, Karagiannis et al. (2002) tested many AR indices and endorsed the Whittle estimator, which takes on values of 0 for white noise, near 0.5 for pink noise, and about 1.0 for brown noise. Named for work by Peter Whittle (1951), a New Zealand/Finnish mathematician, the Whittle estimator was found it to be the most robust in detecting long-range dependence provided that the data are not periodic (Karagiannis et al., 2002; Stadnitski, 2012b), which the movie data are not. We have employed the exact local Whittle estimator (Shimotsu & Phillips, 2005), a further improvement. The Whittle is typically used to estimate the fractional (non-white) noise dimension (*d*) underlying time-series data for autoregression models. Once the nature of this parameter is estimated, other patterns in the data can be explored.^3^

Given the possible contamination of long-term dependence measures by short-term processes and following earlier simulations by DeLong (2015), it occurred to us that the power-spectrum slope values calculated in Study 1 might not be the best estimates of long-range dependence and, hence fractality, in the movie data. Thus, it seemed prudent to re-measure all of our movies with the exact local Whittle estimator.

## Study 2: Shot-duration fluctuations, Whittle estimates, and spectral slopes

Using the Shimotsu and Phillips (2005) version of the Whittle estimator, we calculated the long-term dependence in the full sample of 295 movies of Study 1. Results are shown in Fig. 4b and again the data are noisy. However, there is a slight increase in Whittle value across release years (adjusted *R*^2^ = 0.02, *t*(293) = 2.84, *p* = 0.005, *d* = 0.33), but without a quadratic trend (*t*(292) = 1.7, *p* = 0.09). The linear Whittle trend from 1960 onward is even stronger (*t*(200) = 5.10, *p* < 0.0001, *d* = 0.72). But again, across the whole sample we found a strong correlation between number of shots and the Whittle estimates (adjusted *R*^2^ = 0.15, *t*(294) = 7.14, *p* < 0.0001, *d* = 0.83) as shown in Fig. 4d.

Nonetheless, two further results troubled us. First, although the correlation between slopes and Whittle values for these movies is relatively high (*r* = 0.60, *t*(261) = 12.05, *p* < 0.0001), it is not as high as we had expected. One might attribute this to the difference between the quadratic pattern in the slope data and the linear pattern in the Whittle data. However, as noted earlier, the left-hand side of the quadratic fit is deceiving. It does not, by itself, show a reliable decline. Moreover, whereas the correlation between slope and Whittle values for movies between 1960 and 2015 is reasonable (*r* = 0.57, *t*(200) = 9.86, *p* < 0.0001, *d* = 1.39), that for movies between 1915 and 1955 is no different, it is positive, and it is even marginally higher (*r* = 0.69, *t*(59) = 7.45, *p* < 0.0001, *d* = 1.94). Thus, there is no warrant to worry about the quadratic vs linear regression fits shown in Figs. 4a and b.

Second and more important, after factoring out the effect of shot number on Whittle values, the effect of release year remained a reliable but modest effect (*t*(291) = − 2.35, *p* = 0.02). However, it is in the reverse direction – with smaller Whittle estimates across progressive release years. This latter result suggested that we urgently needed to understand the effects of shot number in movies as measured by both measures – shot spectrum and Whittle estimates. It appeared that Salt (2010) may have been correct, that an increase in shot number alone created fractal-like effects.

## Study 3: Simulations of colored noises at different slopes and vector lengths

### Method

Using the algorithm of Little et al. (2007), we generated 1/*f* ^α^ noises with nine intended alpha values of 0.0 to 2.0 in 0.25-step intervals, and in sample sizes (vector lengths) of 512, 768, 1024, 1536, 2048, and 3072. Intended values are those to which the algorithm should converge in an indefinitely long series. The vector-length range generally conforms to the number of shots in feature-length movies over the century, with the second, fourth, and sixth values halfway between those of 2^9^, 2^10^, 2^11^, and 2^12^. We generated 1000 strings for each intended alpha value at each vector length and measured the actual alpha means and standard deviations of the collection of resulting slopes, using the same algorithm that we used in Study 1 (the modification of that used by Cutting et al., 2010; Gilden, 2001). We did the same for the exact local Whittle estimates (Shimotsu & Phillips, 2005).

### Results and discussion

**Fig. 5:**
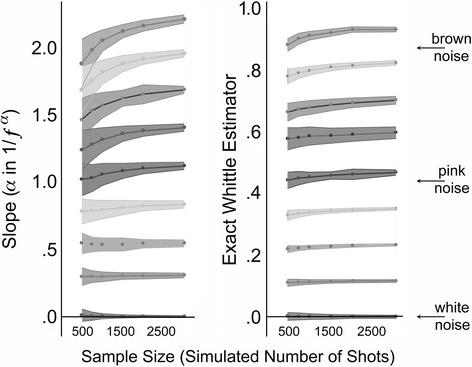
Results of Study 3 investigating the relation between length of vectors and their fractal dimension. The *panels* show means (as points) and standard deviations (not confidence intervals) as shaded areas of noise simulations. Each *point* represents the mean of 1000 simulation trials. Noises were generated by algorithm (Little et al., 2007) and fit by the models used in Study 1 (measuring slope) and in Study 2 (measuring Whittle estimators by the method of Shimotsu & Phillips, 2005). In each panel, noises were generated with intended slopes of 0.0 to 2.0 in intervals of 0.25. Whittle values average about 43% of slope values in these simulations

The patterns of slopes and Whittle estimates as a function of sample size are shown in the panels of Fig. 5, with standard deviations (not confidence intervals) for each function shown in lighter grays. Nine patterns of analysis are shown in each panel, corresponding to results of simulations for intended slopes of 0.0 (at the bottom of each panel) to 2.0 (at the top) at six different vector lengths. Whittle estimates average about 43% of the slope values. To compare the two measures, we rescaled the Whittle estimates, multiplying them by 2.32, so that they had the same grand means. This rescaling value will also be useful in Study 5.

Notice three effects. First, the length of the vector (sample size) does not generally change the measured fractal-like value for power analyses (left panel) with slopes < 1.0 and is not a factor for Whittle estimates for data with slopes approximately < 1.5. However, and second, above these values the length of the vector increased the measured slope. For the overall intended slope values (0.0–2.0) across the six vector lengths, this interaction of some increasing and some non-increasing functions is robust (*t*(100) = 10.81, *p* < 0.0001, *d* = 2.16). Moreover, the increases in slopes with vector length are greater for the power analyses than the increases in the Whittle estimates (a second-order interaction, *t*(100) = 5.59, *p* < 0.0001, *d* = 1.17).

Thus, Salt (2010) was generally correct in suggesting that larger vector strings can generate larger slopes (and even Whittle values). However, the domain in which this increase occurs is not the domain of slopes and Whittle values of the movies investigated here. Again, the mean slope of the 295 movies is 0.55 with a standard deviation of 0.25; the mean Whittle estimate is 0.18 with a standard deviation of 0.10. Both are domains in which the biased inflation of slope values is not apparent.

Third, the standard deviations of the simulated results are relatively smaller for the Whittle estimates than for the power analyses. Setting aside the data for intended slopes of 0.0, the coefficients of variation (standard deviations/means) for the other 48 points (eight intended slopes X 6 vector lengths) are smaller for the Whittle estimates (*t*(47) = 9.04, *p* < 0.0001, *d* = 2.64). Thus, it would appear on the basis of their smaller variation (coupled with the reduced inflationary bias with larger vectors) that the Whittle estimate is a more trustworthy approach to the study of long-range dependence in this context. Moreover, the smaller size of the standard deviations makes it use more appropriate for smaller samples, as we will see in Study 5.

## Study 4: Doubling the shot-duration vector

Although the essentially null results across sample sizes for alphas (slopes) < 1.0 in Study 3 are compelling, the values used to generate them have nothing to do with movies. A comparison of Figs. 1 and 2 shows that there are differences in these waveforms and perhaps some of these are important. To assess the shot-vector case more directly we performed another analysis.

### Method

To provide a second test of a possible effect of vector length on the measured slope and the measured Whittle estimate, we simply doubled the shot vector of each of the 263 movies employed in Study 1 for the power spectra analyses, and did so for the full sample of 295 movies for the Whittle analyses. That is, in each case we concatenated two versions of the shot vector, end to end, the first shot of the repetition abutted to the last shot of the original.

### Results and discussion

**Fig. 6:**
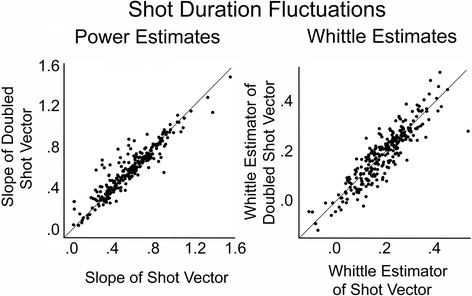
Results of Study 4 where the shot vectors of movies were doubled, concatenated end to end. Slopes (*left*) and Whittle values (*right*) for the doubled shot vectors are plotted against the undoubled results of Studies 1 and 2. The *diagonal lines* represent equal values in both measures; they are not regression lines

Shown in the left panels of Fig. 6 is a scatterplot of movies with the slope values from Study 1 for each plotted against the slope value obtained from doubling the shot vector. The alpha values for the doubled vector are slightly less than those for the original vectors (−0.02) but given the sample size that difference is reliable (*t*(263) = − 3.65, *p* < 0.0001). This effect appears to manifest itself in doubled shot vectors in movies with fewer shots yielding slightly higher values and doubled shot vectors in movies with more shots yielding slightly lower values. Indeed, the slope of the linear regression (not shown in Fig. 6) is 0.87. Thus, there is a small bias in the power spectrum results. Note, however, that the regression values in Fig. 4c for slopes against movies with 750 and 1500 shots is 0.47 and 0.61. Thus, the bias effect here is only about one-seventh of the total effect reported effect in Study 1 and also generally in the opposite direction.

Shown in the right panel of Fig. 6 is the scatterplot of movies with the Whittle values from Study 2 matched to those obtained from doubling the shot vector. Here, too, the mean difference in the two values very slightly disfavors the doubled vectors (− 0.008, regression slope = 0.989), but given the large sample size this modest difference is also statistically reliable (*t*(292) = − 2.94, *p* = 0.004). The magnitude of this difference is about one-ninth of the magnitude of Whittle values for two movies with 750 and 1500 shots – 0.14 and 0.21, taken from the regression line in Fig. 4d – but again in the opposite direction.

One potential flaw in the design of this study is that the exact local Whittle estimator can be sensitive to periodic signals (Shimotsu & Phillips, 2005). Doubling a shot vector certainly introduces periodicity. However, parallel simulations were also done for enantiomorphic doublings of the shot vector (*z*-to-*a* concatenated to the end of *a*-to-*z*) and the results were indistinguishable from those seen in the right panel of Figs. 6.

Thus, the results of both Studies 3 and 4 strongly suggest that one of the results of Studies 1 and 2 – the increase in long-range dependence shown in the shot-duration vectors in more recent films – is, contrary to Salt (2010), not simply a result of a movie having more shots. Instead, they must have another cause. Something has been done to the shots in the crafting of the movies as the shot strings got longer over time. As Cutting et al. (2010) suggested one likely candidate of this cause is the movie editor, although other filmmakers would certainly play important roles.

To explore the idea that filmmakers might craft these long-range effects, we needed more dimensions of movies to consider. Study 5 looks at the durations of scenes within a movie, motion within shots, and sound amplitude within movies. Study 6 looks at the properties of luminance, clutter, and shot scale within shots. Full disclosure: We sorted those dimensions into the two studies after we knew their results.

## Study 5: The changing fractal-like patterns of scene durations, motion, and sound amplitude

Across the studies done in our lab, we have measured much more than just shot durations of movies. Using and expanding on our analyses of scenes, motion, and sound here – and luminance, clutter, and shot scale in Study 6 – we set out to explore any possible fractal-like progressions that might be relevant and irrelevant to what filmmakers do in fashioning movies. As before, we normalized the values in each of the six types of arrays and calculated their exact local Whittle estimators.

### Method

#### Scenes and scene durations

Typically, with cuts at either end, a shot’s duration is easy to measure. But what is a scene? A scene in theater is typically defined as an event that takes place in one location, over a contiguous stretch of time, with a fixed set of characters (Polking, 1990, p. 405), and the same typically holds for movies. A scene boundary occurs with a change in one or more of these three attributes. Moreover, a scene can typically be said to have a beginning, middle, and an end – an integrity that creates a whole. Movies can also have something different, which we call subscenes. These have changes in location, time, and character but they often have neither beginnings nor ends. They are essentially ongoing middles and they signify parallel action. This technique is called cross-cutting. Cross-cutting has been used since the beginning of feature-length movies in the 1910s and it has generally increased over time. For example, the climax of action films often cross-cuts between the protagonist and antagonist in different locations before they meet in a final confrontation. Here, as we have in past studies (Cutting, 2014a; Cutting, Brunick, & Candan, 2012), we combine the analyses of scenes and subscenes, and simply call them scenes.

Cutting et al. (2012) employed a subsample of 24 movies from the larger sample – three genres (a drama, a comedy, and an action film) at eight release years (1940, 1950, 1960, 1970, 1980, 1990, 2000, and 2010). They had three observers watch each movie twice, first simply to enjoy it and second to segment the film into scenes and record the frame number of the beginning of the scene. All segmentations that were agreed upon by at least two of the observers were used here and the duration of the scenes measured. These movies average 106 scenes but, as will be discussed below, their number varies by release year (Cutting, in press). The scene durations were normalized as in the previous studies and the scene-duration vector was analyzed using the exact local Whittle estimator, as in Studies 2–4.

#### Motion

To analyze motion (and luminance and clutter in Study 6), we eliminated some movies from consideration. It seemed unwarranted to look at the motion, luminance, and clutter profiles of silent movies – those in this sample from 1915 to 1925. In total, 10–20% of all shots in these movies are intertitles. Intertitles have no motion (except jitter from low-budget analog-to-digital transfer), are typically black, and are cluttered only with white text. We also did not have these data by shots for the children’s movies (Brunick, 2014) or the two used by Cutting, DeLong and Brunick (2011). This left 180 movies in the sample for measures of motion, luminance, and clutter. These movies were released from 1930 to 2015.

Motion can be measured in many ways but in natural stimuli all tend to be strongly correlated (Nitzany & Victor, 2014). Thus, we have chosen the simplest, which can be called zero-order motion. We measured the correlation of pixels in one image with those of the next (actually the next adjacent, n and n + 2, to reduce digitization artifacts and avoid an issue in earlier animated movies where frames are often doubled). At 24 frames/s the average movie in this sample has 155,000 frames. This method of motion measurement was used by Cutting, DeLong and Brunick (2011) and Cutting (2016a, 2016b). We had first downsampled each frame of the movies to a 256 × 256 array (about 65,000 pixels), then converted color frames to 8-bit grayscale (with pixel values in the range of 0–255), correlated the frames, and then averaged all across-frame correlations within each shot (but not those including frames straddling a cut). This value determined the motion within that particular shot. Variations of the within-shot correlations across time, and hence the variation in amount of motion, are shown in Fig. 2b for the first 512 shots of *Dances with Wolves* (Kostner, 1990).

#### Sound amplitude

Here we employed 48 movies used previously by Cutting (2015), three movies per release year divisible by five from 1935 to 2010 – one drama, one comedy, and one action film. The appendix of Cutting (2015) lists those movies and 24 of them were used by Cutting et al. (2012). A sample waveform is shown in Fig. 2c for the sound pattern in the Marx Brothers film, *A Night at the Opera* (Wood, 1935).

Shots are visually discontinuous across their boundaries, but audio is not; it flows smoothly across cuts and helps to make cuts less apparent to viewers (Shimamura, Cohn-Sheehy, Pogue, & Shimamura, 2015). Thus, we ignored shots in this analysis. From the video files, we extracted the audio track at a sampling rate of 44,000 Hz. We then divided the length of the movie into 100-frame bins (4.17 s) and assessed the amplitude of the combined stereo tracks. We then created a bin vector for fractional analysis, which varied by the length of the movie.

### Results and discussion

#### Scene durations

**Fig. 7:**
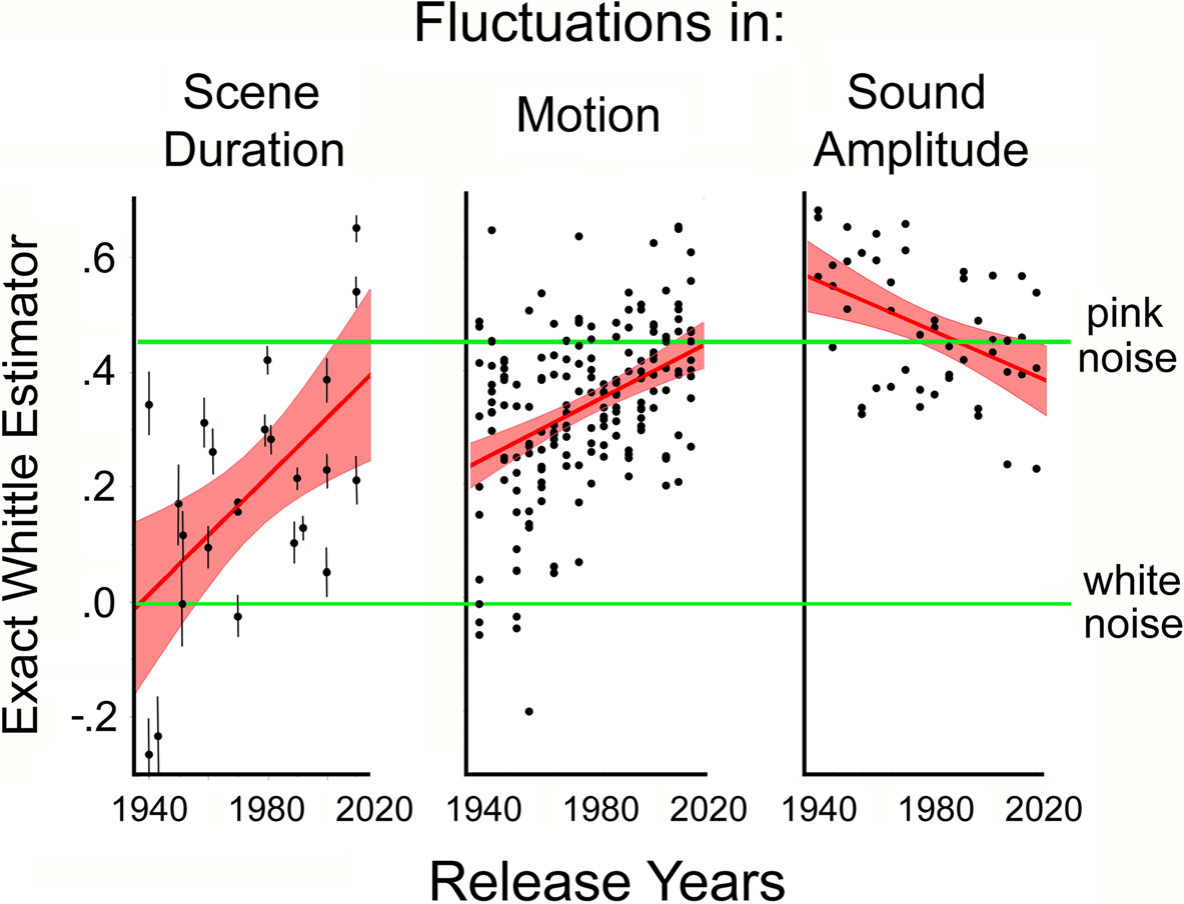
Results of Study 5 plotting fractal dimension against release years for three movie variables. *Left:* A *scatterplot* of the increase in long-range dependence, measured by the exact local Whittle estimator and of the scene-duration vectors of 24 movies from 1940 to 2010. The regression line and 95% confidence intervals are also shown. The small *error bars* in the *left panel* indicate the standard deviations in generating 1000 pseudo-random sequences with the measured Whittle value and number of scenes for the given movie. *Middle:* The scatterplots of the fractal-like measure of motion in each shot for the shot vector in 180 movies. *Right:* A decline in long-range dependence for sound amplitude in sample vectors of 48 movies. All panels show a reliable change in long-range dependence over release years. The *upper horizontal green line* represents the approximate fractal value of 1/*f* ^1^ (pink noise) as determined in Study 3; the *lower line* represents 1/*f* ^0^ (white noise)

As with the fluctuations of shot durations, there has been increasing long-range dependence in the duration patterns of scenes in movies released over the last 70 years. The Whittle estimates by release year are shown in the left panel of Fig. 7, along with their regression line (adjusted *R*^2^ = 0.29, *t*(22) = 3.26, *p* = 0.004, *d* = 1.40) and the 95% confidence interval. Notice that the Whittle estimates show a striking increase. For scenes in contemporary movies the values very nearly reach 0.43, which is analogous to a fractal (1/*f* ^1^) value in these data, having started as nearly white noise in 1940.

Stadnitski (2012a) recommended that at least 500 observations be used before estimating *d* (for dimension) in autoregression or power analyses, whether by Whittle values or any other method. The number of scenes in these 24 movies fall well short of that recommended criterion. Thus, other calculations are needed. The left panel of Fig. 7 also shows error bars on the datum for each movie. These are standard deviations and were determined using an analog to the procedure in Study 3. The Whittle estimate (*d*) of each movie’s scene pattern was converted to an intended alpha value (α ≅ 2.32*d, as determined in Study 3) and 1000 random time series were generated for that intended alpha and the number of scenes in that movie. Whittle standard deviations were recorded, which were in the range of 0.12–0.02 for vectors between 35 and 215 scenes. Results show that the shortness of the scene-duration vectors does not contribute markedly to the results in the left panel of Fig. 7.

With these results, we now know that the increase in long-range dependence across release years can be found in both the fluctuations of shot durations and scene durations. Moreover, these occur at different scales. The mean, median, and modal number of shots per scene are 11 shots, six shots, and one shot, respectively. The mean, median, and modal shot durations in the 24 movies sampled here are 5.5, 3.1, and 1.5 s, respectively; and the mean, median, and modal scene durations are 81, 57.5, and 12.5 s, respectively. Moreover, and somewhat surprisingly, the Whittle estimates for the shot-duration fluctuations and the scene duration fluctuations across movies are negatively correlated, although not strongly so (*r* = − 0.24, *p* = 0.26). Thus, the shot-duration fluctuations are not merely a subset of those for scene durations, nor are the scene-duration fluctuations a superset of those for shots. They are simultaneous, fractal-like patterns at different and offset scales.

#### Motion

The central panel of Fig. 6 shows the motion data with a rising trend across all release years (adjusted *R*^2^ = 0.17, *t*(178) = 6.01, *p* < 0.0001, *d* = 0.90). The rise for movies from 1960 onward is also reliable (adjusted *R*^2^ = 0.032, *t*(118) = 2.24, *p* = 0.027, *d* = 0.41). This function is not as steep as that for scenes, but is steeper than that in Fig. 4b for the shot-duration data. Moreover, like that for scenes, it converges on fractal values in the contemporary movies of this sample (again, the 1/*f* ^1^ simulations of Study 3 generated Whittle values near 0.43). Moreover, this effect is linear; there is no hint of a quadratic trend (*t*(176) = 0.77, *p* = 0.44).

#### Sound amplitude

The right panel of Fig. 6 shows something unusual in this context – a downward sloping function across release years (adjusted *R*^2^ = 0.18, *t*(46) = − 3.41, *p* = 0.0014, *d* = 1.01), again without a hint of a quadratic function. We were quite surprised by this finding, but we take it as strong evidence that something like fractal (pink noise) variation is likely the implicit target goal of the filmmakers (in this case likely the sound editor), not just something that increases as one massages the dimensions of a movie to try to improve it.

## Study 6: The unchanging fractal-like patterns of luminance, clutter, and shot scale

Among the other physical measures of movies that we have recorded and investigated are the mean luminance of each shot, the mean clutter in each shot, and the mean shot scale (see Cutting, 2016a, 2016b; Cutting & Armstrong, 2016, 2018). So, we focus in this study on these patterns of long-range dependence across release years.

### Methods

#### Luminance

Again, frames from 180 movies (used for motion analysis in Study 5) were downsampled to 256 × 256 arrays. Color movies were converted to grayscale, then all frames in all movies were gamma corrected, and the median value taken for all pixels in each frame. For all frames within a shot, the average of those medians was taken. Variations in the mean shot luminances are shown in Fig. 2d for the first 512 shots of *Westward Ho* (Bradbury, 1935).

#### Clutter

We used a method from Rosenholtz et al. (2007; see also Cutting & Armstrong, 2016; Henderson et al., 2009), adapted from static images. Here, for the same 180 movies we took every tenth downsampled frame within each shot and passed it through a Laplacian of Gaussian filter, a standard edge detecting algorithm (see Marr, 1982, pp. 58–59). This creates a mostly black image with jagged, single-pixel-width white lines corresponding to the edges in the original image. We then counted the percentage of white pixels in the otherwise black image, averaged those values within a shot, and that averaged proportion was our estimate for the clutter of each shot. The assumption here is that more edges equal more objects and textures, equals more clutter. A clutter waveform of the first 512 such shots in *Superman II* (Lester, 1980) is shown in Fig. 2e.

#### Shot scale

For the third measure of this group, we returned to the 24 movies from Study 5 released from 1940 to 2010 (Cutting et al., 2012). For each of those movies we had categorized the scale of each shot, which is essentially the measure of the size of the head of a character in the frame. Conventionally, this is done allocating shots to a seven-point scale, although the measure is actually continuous. In this context, 1 = an extreme long shot (usually of landscapes, cityscapes, or seascapes in which, if there are any characters visible, they are quite small); 2 = long shot (where the full body of the character can be seen, but there is little space in the frame above her head or below her feet); 3 = medium long shot (the character is seen above the knees); 4 = medium shot (above the waist); 5 = medium closeup (chest up); 6 = closeup (head and shoulders); and 7 = extreme closeup (face or part of a face only, or a shot of a comparably sized object). A sample waveform is shown for *Star Wars: Episode 5 – The Empire Strikes Back* (Kershner, 1980) in Fig. 2f. Notice the effect of the quantized 7-point scale.

### Results and discussion

#### Luminance and clutter

**Fig. 8:**
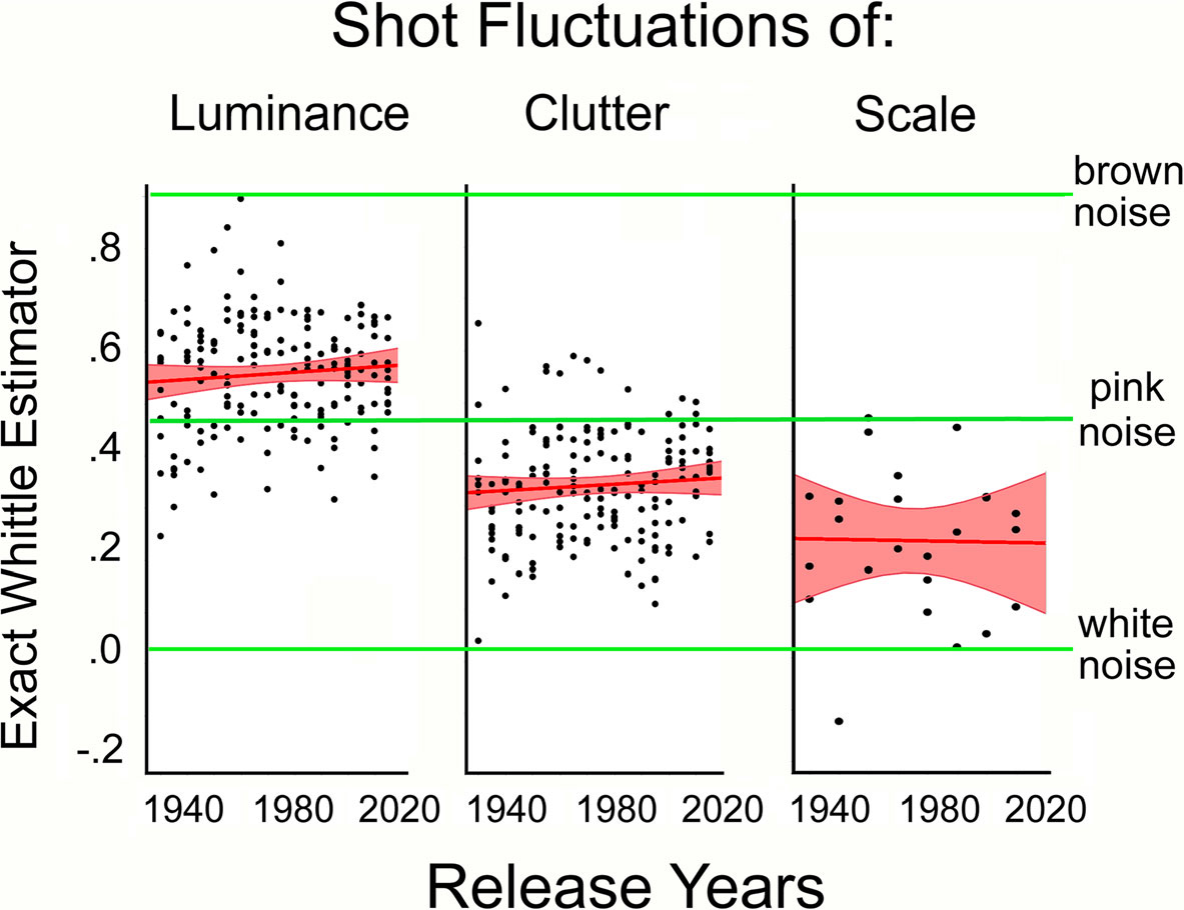
Results of Study 6 for Whittle estimates of three dimensions in the shots of movies. *Left:* The unchanging Whittle values for shot-luminance vectors across release year for 180 movies. *Middle:* The same measure for shot-clutter vectors for 180 movies. *Right:* Shot-scale vectors in 24 movies. The *upper horizontal green line* represents the approximate value of 1/*f* ^2^ (brown noise) as determined in Study 3, the *middle line* represents the approximate value of 1/*f* ^1^ (pink noise), and the *lower line* the value of 1/*f* ^0^ (white noise)

The panels of Fig. 8 show quite different results from those of Fig. 7. The left panel shows an essentially flat function for the luminance data (adjusted *R*^2^ < 0.01, *t*(178) = 1.12, *p* = 0.26). The mean Whittle values for the luminance data are 0.56, quite a bit above a pure fractal. The likely reason for this is that luminance changes across shots within a scene are a bit like Brownian motion, as seen in the right panel of Fig. 1. Dominating the pattern in Fig. 2d are random and small deviations in shots from a pedestal value for the scene, but then these are followed by sometimes-large changes in luminance across scenes. The middle panel of Fig. 8 shows a similar flat function for the clutter data (adjusted *R*^2^ < 0.01, *t*(178) = 1.0, *p* = 0.32). The mean Whittle values for clutter are different, 0.34, and a bit closer to pink noise (*d* ~ 0.43) than to white noise (*d* ~ 0.0).

Importantly, both luminance and clutter fluctuation patterns across release years from 1930 to 2015 contrast with that of motion, shown in the central panel of Fig. 7. The interaction with release years is strong for both the motion-luminance comparison (adjusted *R*^2^ = 0.09, *t*(177) = 4.29, *p* < 0.0001, *d* = 0.64) and for the motion-clutter comparison (adjusted *R*^2^ = 0.10, *t*(177) = 4.46, *p* < 0.0001, *d* = 0.67).

#### Shot scale

Finally, right panel of Fig. 8 shows the results for the much smaller array of shot scale fluctuation data. Again, the data are noisy and there is no clear pattern across release years (adjusted *R*^2^ < 0.01, *t*(22) = − 0.08, *p* = 0.94). Mean Whittle values are 0.22, about halfway between white noise and a fractal value. Given the differences in sample size we cannot directly compare these results with those of motion, but we can compare them against the scene results in the left panel of Fig. 7. Importantly, the interaction of the difference in Whittle values for scenes and shot scales across release years is considerable (adjusted *R*^2^ = 0.20, *t*(22) = 2.53, *p* = 0.02, *d* = 1.08).

## Caveat: Changes in means vs changes in fluctuation patterns

At this point it is important to keep in mind two kinds of trends within movies across release years – those of the dimensions (reflected in changes in means over time) and those of the fluctuations in those dimensions (reflected in changes in the long-range dependence measure over time). The latter are the focus of this article, but our lab has previously investigated the former in detail. Consider again six dimensions – shot duration, scene duration, motion, luminance, clutter, and shot scale. We had no previous estimates of the seventh dimension – mean sound amplitude – across years, and given the changes in technology and theater presentation we would find it difficult to suggest anything useful.

### Shot duration

Perhaps the most widely reported change in popular movies is in their shot durations over time (see Bordwell, 2006; Cutting, 2015; Cutting, DeLong, Brunick, Iricinschi, & Candan, 2011; Salt, 2006, 2009). Shots have generally gotten shorter, decreasing more or less linearly from a mean of about 10 s/shot in 1950 to about 4 s/shot in 2010. As noted by Cutting et al. (2010) and as found in Studies 1 and 2, the shot-duration fluctuation patterns have also changed. But in contrast, these have generally increased in their long-range dependence over the same period.

### Scene duration

Scenes have also changed in these two ways. As noted by Cutting et al. (2012) and as suggested in the results of Study 5, scenes have generally gotten shorter over time, decreasing from a mean of about 90 s/scene in 1940 to that of about 50 s/scene in 2010. But the fluctuation patterns, as measured in Study 5 by the Whittle estimate for long-range dependence, have generally increased over this period.

### Motion

Amounts of motion have also changed across release years. Cutting, DeLong, Brunick, Iricinschi, & Candan (2011) reported that the mean motion in shots, averaged across the length of movies, has increased from 1935 to 2010. Study 6 here found that the measured long-range dependence in the fluctuation patterns of motion in movies has also generally increased, here from 1930 to 2015.

### Luminance

Cutting et al. (2011) reported that the mean luminance in shots has decreased from 1935 to 2010, due in part to increased sensitivity of film stock and the later conversion to digital formats. This change also increases contrast, allowing greater luminance differences between brighter objects (and people) and darker backgrounds (Cutting, 2014b). However, Study 6 showed that the long-range dependence in fluctuation patterns of luminance across the lengths of movies has been quite varied but is unchanged over roughly this same period.

### Clutter

One measure of the complexity of the cinematic image has also shown change. We have not previously published data on the mean clutter per shot before but these have decreased more or less linearly from 1930 to 2015 (adjusted *R*^2^ = 0.10, *t*(178) = − 4.55, *p* < 0.0001, *d* = 0.68), with the individual frames of movies becoming “cleaner.” That is, in more contemporary movies, there tend to be fewer people (Cutting, 2015), fewer objects, and fewer textures in the background that might distract the viewer. Yet Study 6 showed that the long-range dependences in the fluctuation patterns of clutter in shots is unchanged over the same period.

### Shot scale

The standard measure of the size of the character in the movie frame has shown a change across time. Salt (2006, 2009), Cutting and Armstrong (2018), and Cutting et al. (2012) have shown that the size of the character’s head in movie frames has increased in movies from the 1910s to 2010. In silent film and early sound film, the average shot scale across a whole movie was a medium long shot (showing the character from knees up) whereas, as cameras got smaller and more mobile, by 2015 it was closer to a medium closeup (showing the character from the chest up). This change makes the character’s facial expressions and emotions easier to discern. In contrast, Study 6 showed that the fluctuation patterns in shot scale across movies has essentially remained unchanged between 1940 and 2010.

It is also important to remember that the changes in the means of these dimensions over time can have no influence on their measured fluctuations. All dimension values are normalized before the fluctuations were measured (means = 0, standard deviations = 1). Thus, the absolute durations of shots and scenes, and the extent of motion, the amounts of luminance and clutter, and measures of shot scale can have no role in results showing long-range dependence and their possible changes over time.

## General discussion

We now have seven datasets assessing release-year trends of long-range fluctuations in movie sequences. Post hoc, we find two groups. The first group consists of fluctuations of shot durations, scene durations, shot motion, and sound amplitude all converging on a fractal value over at least 70 years from the 20th and into the 21st century. The second group consists of shot luminance, shot clutter, and shot scale, all of which show no trends over time. What might we be able to say about why these dimensions differ? And why have fractal patterns (long-range dependence) emerged in one set of them? Let us begin with statements by filmmakers themselves.

### Pulse, pace, and the intentions of filmmakers

Every popular movie is made by a large team of individuals and it is difficult to assign credit to any one member for any one dimension of a film. To simplify matters, however, let us concentrate on the jobs of four individuals or small groups, roughly in the order they come to the task of filmmaking – the scriptwriter, the director, the cinematographer, and the editor. These are the primary individuals that control the timing, the dynamics, and the energy of the movie.

Consider four views on their roles in successful storytelling that might be relevant to our time series results. Scriptwriter Aronson (2000, p. 40) believes that film structure is about good timing. She states:

In fact everything about film – about *moving pictures* – is connected with time and movement in time, that is to say action, in every sense. Film consists of movement in all ways, physical, emotional, and spiritual. In screenwriting, story is movement and our characters move through their own mental landscapes (italics in the original).

Directors are busy with a multitude of jobs and seem not to write about their role in the shaping the cadence of movies. However, New York city theater director Parker (2012) echoes Aronson’s view and expresses part of her role in terms that apply equally to a movie director.

good direction can be spotted in transitions… The director is the lynchpin of pace, because it’s a thread that goes through every part of the production.

Cinematography means “motion writing,” and cinematographer Brown (2012, p. 210) writes about similar goals:

moving the camera is much more than just going from one frame to another. The movement itself, the style, the trajectory, the pacing, and the timing in relation to the action all contribute to the mood and feel of the shot.

And finally, film editor Pearlman (2009, pp. 63–64) is fundamentally interested in the perception of rhythms. Paramount to her are:

the functions of rhythm in creating cycles of tension and release and synchronizing the *spectator’s* rhythms to the film’s pulse and its fluctuations … the art of shaping rhythm is a choreographic art in that it involves shaping physical movement for affect. The core unit of this choreographic art is pulse. (italics in the original).

Thus, among the many other aspects of making a movie or a play, these individuals point to the creative goals of finding and making the appropriate dynamics, or pulse.

### What’s attention got to do with it?

Cutting et al. (2010) linked their results to fractal patterns in human cognition (Gilden, 2001, 2009), but at the time the linkage seemed tenuous. Can we now do better? According to Pearlman (2009), the goal of the filmmaker is to create sensory, perceptual, and emotional rhythms in a movie and to synchronize the viewers’ rhythms to them. We now know that there are fluctuations in the shot duration, scene duration, motion, and sound patterns in movies that have become more fractal-like over time. We also know that audience eye movements (Hasson et al., 2008) and brain activation patterns (Hasson et al., 2009) are synchronized with one another and with the content of the movie. We know further that movie shots typically are joined across a cut and that cuts trigger eye movements (Mital, Smith, Hill, & Henderson, 2011, Smith 2013), a low-level demand for attention. And we know that eye movements during visual search in the laboratory typically have a fractal pattern (Aks, Zelinsky, & Sprott, 2002).

Cuts, however, are often cognitively disguised by continuity editing (Smith, 2012). That is, despite what their eyes are doing, viewers, when given the overt task to detect cuts, miss many of them entirely (Smith & Henderson, 2008). Indeed, editors typically try to make cuts cognitively invisible (Shimamura et al., 2015) by the technique called matching-on-action. Leftward motion in one shot is matched by leftward motion in the next. Sudden changes in direction of motion are known attract attention (Howard & Holcombe, 2010; von Mühlenen & Lleras, 2007), which is the likely reason why editors match-on-action in shots within scenes. Cuts separating scenes, on the other hand, are rarely missed (Smith, Levin, & Cutting, 2012). Brain activity is quite different at scene boundaries as opposed to shot boundaries within a scene (Magliano & Zacks, 2011; Zacks, Speer, Swallow, & Maley, 2010).

Finally, it is very important for viewers to register scene boundaries to track the narrative (Sargent et al., 2013). Otherwise comprehension and memory are jeopardized. This process is called event segmentation, which is quite automatic and happens largely under exogenous control (Zacks & Swallow, 2007) – that is, determined by the movie. Moreover, failures of event segmentation in the real world can be a sign of cognitive decline (Richmond, Gold, & Zacks, 2017).

Thus, when we as movie viewers track shots, scenes, and their content in a contemporary film (and we must), we are paying attention to fractal-like patterns. When we move our eyes after a cut (and we reflexively do), we are responding to a larger a fractal-like pattern. When we respond to the motion across shots (and we instinctively do), we are also tracking a larger fractal-like structure. And when we listen to the sound pattern of a movie (and we mandatorily do), we are following temporal fractals. And again, we know that the goal of filmmakers is to have viewers respond to the fluctuations that they create (Pearlman, 2009, 2017) and that viewers’ eyes and minds respond to those fluctuations (Hasson et al., 2008, 2009). So, that’s what attention has to do with it.

### Fractal convergence and non-convergence

We began this article with the claim that fractal patterns are nearly ubiquitous. This statement is a bit misleading. Instead, we believe that random patterns are incredibly rare and that in the temporal domain there is a wide array of naturally and socially occurring patterns between and beyond 1/*f* ^0^ and 1/*f* ^2^. Temporal fractal patterns, those near 1/*f* ^1^, can be particularly interesting, in part, because they are found in a wide variety of human processes from physiology to cognition. The dimensions of movies that converge on fractals – shot durations, scene durations, motion, and sound amplitude – can be argued to be important to the viewer’s attention. Image changes (transients cause by cuts), motion changes (with and across shots), and sound changes attract attention. In addition, scene changes are important for comprehension. Thus, it is not difficult to imagine why filmmakers might craft these dimensions if fractality is an implicit goal.

But what about luminance, clutter, and shot scale? Are these dimensions less important in holding viewers’ moment-to-moment attention. We know that luminance changes can grab attention (Cole, Kuhn, & Skarratt, 2011; Spehar & Owens, 2013; Theeuwes, 1991), but film luminance is a more a property of a scene than it is of the shots that make it up. That is, luminance is less likely to change across shots within a scene than across shots at a scene boundary. Moreover, it is only a weak cue to scene change – only 40% as potent as color change and only 4% as potent as time or location changes taken together (Cutting et al., 2012). It is certainly true that luminance, like color, affects mood and tone in a movie, but it does not seem as if luminance change would be a frequent driver of attention in a movie. Perhaps that is a reason why filmmakers have not systematically changed their use of it over time.

Clutter has been important to popular filmmakers over time. Earlier, we reported that the clutter in movie images (where clutter is a proxy for the number of objects, people, and textures) has decreased over the last 90 years. The reason for this, it seems clear, is to narrow the focus of the viewer, removing extraneous objects and people from the image, to control gaze more effectively. Shots will vary in their clutter, but clutter distracts attention rather than attracting it. Thus, there would seem to be no reason for filmmakers to manipulate it to help assure their viewers are engrossed in a movie.

Finally, shot scales do vary systematically across a scene and their variation tends to have a basic scalloped profile. Scenes tend to begin with a long or medium-long shot, then the camera moves in on the characters as they converse, and then it often backs off at the end of the scene (Cutting et al., 2012). The major purpose of this pattern is, in the first shot to introduce the viewer to the surroundings of the characters and their relative positions within it, in the next shots to focus better on the conversationalists and particularly their faces so the viewer can discern their emotional states (Cutting & Armstrong, 2018), and then often to back away. Change in shot scale is a strong signal for scene changes, seven times more potent than luminance changes (Cutting et al., 2012), and thus is a candidate for grabbing the viewer’s attention. Thus, it could serve the effect of fractals in scene durations, but since shot scale has little to do with duration (but see Cutting & Armstrong, 2016) it need not be manipulated by filmmaker’s other than to structure scenes.

We recognize that our accounts of the non-changes in the fractional dimension of some attributes of movies may seem ad hoc. We also recognize that our accounts of the psychological effects of the fractal confluences of other attributes are only based on correlations. Nonetheless, we find our results and these possible accounts compelling, and possibly even true.

### Thoughts on the emergence of fractals in movies

Pearlman (2009) wrote that the pulse of movies is central to engrossing viewers. It is worth remembering that pulse is not a metronomic beat. The three most important pulses for human beings are those of the heart, the lungs, and the feet. We know that healthy heart beats (Goldberger et al., 2002), healthy respiration (Hoop, Burton, & Kazemi, 1996), and healthy gait (Hausdorff, 2007) all have a fractal pulse. In contrast, evenly spaced heartbeats and breaths are symptoms of disease and evenness of gait is a prediction for an upcoming fall. Health has been described as coordination of multiple systems and subsystems working at different time scales. The flexible and adaptive interactions of these systems are the very basis of health, and the form of their interaction is a balance of competitive and cooperative processes (Van Orden, 2007). So, a first idea is that this seems like an appropriate metaphor for the teamwork needed in creating a movie – cooperation and competition among filmmakers, working together but trying to do their best as they see fit, manipulating the dimensions that they see fit.

A second idea comes from the only other domain that we know of in which time-series data have converged on fractal values over time. That domain is motor control, where fractal-pattern results are relatively commonplace in the literature. Using a large digitizer tablet, Wijnants et al. (2009) trained individuals to draw lines with their non-dominant hand back and forth between two targets as rapidly and accurately as possible. They then treated as time-series data the between-target movement times of 1100 trials in each of five blocks. Across blocks, mean reaction time declined and mean slope increased to 1/*f* ^1^. Thus, well-practiced individuals generate fractal patterns whereas the same individuals, when unpracticed, generated approximations closer to white noise.

Could the achieved coordination in motor control be analogous to a recently achieved, smoother coordination of filmmakers in producing a movie? Could it be that the cultural inculcation and cross-generational acquisition of skills by filmmakers over decades is like the motoric practice of single subjects over minutes? Have filmmakers’ products converged on fractals because of their attained increase in skill and fluidity? Such an account is speculation, but a tantalizing one.

## Summary and conclusion

The results of Studies 1 and 2 extend those of Cutting et al. (2010). The fluctuating patterns of shot durations in movies released over the past century are approaching a fractal pattern. But there are two caveats. First, that approach has been slow (Study 1) and, second, it is clear that measuring the slope of the power spectra appears not to be the best way to assess the long-range dependencies in the data (Study 2). Exact local Whittle estimators seem better. Nonetheless, the Whittle estimate results tend to reinforce the data and conclusions of Cutting et al. (2010).

Results also showed that slopes and Whittle values have increased with the number of shots in movies (Studies 1 and 2). However, shot-vector lengthening is not causally related to that increase, and thus is not a measurement artifact (Studies 3 and 4). Longer arrays do not inherently produce increased spectral slopes or increased long-range dependence; filmmakers do.

The results of scene duration fluctuations, motion fluctuations in shots, and sound amplitude fluctuations (Study 5) also show changes in long-range dependence over time, all converging on fractal values, two from nearer white noise and one from nearer brown noise. Together, these results suggest that fractality has been an implicit target in the evolution of temporal factors in movies. Movies may engross viewers, in part, by synchronizing them to its fractal patterns. This may be one of the reasons movies are so engaging and so popular worldwide.

A century ago, Münsterberg (1915) suggested that psychologists should study movies to study the mind. Fortunately, psychologists, neuroscientists, and others are now doing so. Movies, because of their multimodal complexity and their ability to place us in varied emotional and cognitive states, are a nearly bottomless fount for psychological research. Happily, we can now carry out Münsterberg’s wishes.

## Footnotes

1 For a sample misconstrual about our study and attention span see Kaliym Islam (1 Mar 2013) “Attention span and performance improvement,” Ariel, https://www.trainingindustry.com/blog/performance-management/attention-span-and-performance-improvement/; and for a sample misinterpretation about a link between 1/*f* and the quality of films see Jeremy A. Kaplan (26 February 2010) “The science of Hollywood films: It’s all in the chaos theory,” FoxNews.com, https://news.nationalgeographic.com/news/2010/02/100225-foxnews-science-of-hollywood-films-chaos-theory/.

2 See the eight blogposts in the section entitled “Shot lengths and psychology: Has attention to do with it?” at http://www.cinemetrics.lv/articles.php. Most of these are concerned with discussions of the short-range autoregression measures used by Cutting et al. (2010), a topic beyond the scope of this article.

3 Although it is not our focus, the goal of AR models is prediction. AR models assume that, given a string of values (a time series), the upcoming value is best predicted by a combination of two factors: (1) the pattern underlying the previous values; and (2) a stochastic (noise-generating) process also reflected in those values. There are many types of AR models, one of which is ARFIMA (autoregressive fractionally integrated moving average). The term “fractionally” refers to the fractional (fractal-like) dimension, *d*, of the noise: 0.0 = white noise, 0.5 = pink noise, 1.0 = brown noise, and possible noises in between and beyond. The parameter *d* is one of the inputs into ARFIMA model. Thus, the value of *d* captures the nature of the noise generated that accompanies the pattern of typical interest. Here, however, we are not interested in the possible underlying pattern in the time series (but see Cutting, 2016a, 2016b); we are interested only in the “noise” itself. An estimator of that noise is based on an algorithm that is used to characterize its distribution, expectancy values, and variances – all captured by the estimated fractional value, *d* (Stadnitski, 2012a). Estimators come in a number of varieties and the Whittle estimator belongs to the family based on periodograms (the other two are based on maximum likelihoods and detrended fluctuation analysis). Periodograms are similar to Fourier analysis but optimized for unevenly time-sampled data and differently shaped basis functions. Thus, the Whittle would appear to be more flexible in fitting noise data than our spectral analysis. This flexibility could be reflected in the difference in standard deviations in the results of Study 3 (Fig. 5).

## References

[CR1] Aks DJ, Zelinsky GJ, Sprott JC (2002). Memory across eye-movements: 1/*f* dynamic in visual search. Nonlinear Dynamics, Psychology, and Life Sciences.

[CR2] Aronson, L. (2000). *Screenwriting updated: New (and conventional) ways of writing for the screen*. Los Angeles: Silman-James Press.

[CR3] Bezdek MA, Gerrig RJ, Wenzel WG, Shin J, Pirog Revill K, Schumacher EH (2015). Neural evidence that suspense narrows attentional focus. Neuroscience.

[CR4] Bordwell D (2006). The way Hollywood tells it.

[CR5] Bordwell D, Staiger J, Thompson K (1985). The classical Hollywood cinema: Film style & mode of production to 1960.

[CR6] Brown B (2012). Cinematography: Theory and practice.

[CR7] Brunick, K. L. (2014). *Low-level perceptual features of children’s films and their cognitive implications* (Ph.D. Dissertation). Ithaca, NY: Cornell University. http://hdl.handle.net/1813/38853. Accessed 1 Sept 2017.

[CR8] Cole GG, Kuhn G, Skarratt PA (2011). Non-transient luminance changes do not capture attention. Attention, Perception & Psychophysics.

[CR9] Cutting, J. E. (2014c). More on the evolution of popular film editing. http://www.cinemetrics.lv/dev/cuttingcinemetricx3.pdf. Accessed 1 Sept 2017.

[CR10] Cutting, J. E. (in press). Simplicity, complexity, and narration in popular movies. In M. Grishakova & M. Poulaki (Eds.) *Narrative complexity and media: Experiential and cognitive interfaces*. Lincoln, NE: University of Nebraska Press.

[CR11] Cutting JE (2014). Event segmentation and seven types of narrative discontinuity in popular movies. Acta Psychologica.

[CR12] Cutting JE (2014). How light and motion bathe the silver screen. Psychology of Aesthetics, Creativity, and the Arts.

[CR13] Cutting JE (2015). The framing of characters in popular movies. Art & Perception.

[CR14] Cutting JE (2016). Narrative theory and the dynamics of popular movies. Psychonomic Bulletin & Review.

[CR15] Cutting JE (2016). The evolution of pace in popular movies. Cognitive Research: Principles and Implications.

[CR16] Cutting JE, Armstrong KL (2016). Facial expression, size, and clutter: Inferences from movie structure to emotion judgments and back. Attention, Perception & Psychophysics.

[CR17] Cutting JE, Brunick KL, Candan A (2012). Perceiving event dynamics and parsing Hollywood films. Journal of Experimental Psychology: Human Perception and Performance.

[CR18] Cutting JE, DeLong JE, Brunick KL, Iricinschi C, Candan A (2011). Quicker, faster, darker: Changes in Hollywood film over 75 years. i-Perception.

[CR19] Cutting JE, DeLong JE, Brunick KL (2011). Visual activity in Hollywood film: 1935 to 2005 and beyond. Psychology of Aesthetics, Creativity, and the Arts.

[CR20] Cutting JE, DeLong JE, Nothelfer CE (2010). Attention and the evolution of Hollywood films. Psychological Science.

[CR21] Cutting, J. E. & Armstrong, K. L. (2018). Cryptic emotions and the emergence of a metatheory of mind in popular filmmaking. *Cognitive Science.*10.1111/cogs.12586. 10.1111/cogs.12586 PMC6001644 29356041

[CR22] DeLong, J. E. (2015). *Time series analysis of Hollywood film and reaction times* (Ph.D. Dissertation). Ithaca, NY: Cornell University. http://hdl.handle.net/1813/39455. Accessed 1 Sept 2017.

[CR23] Farrell S, Wagenmakers E-J, Ratcliff R (2006). 1/*f* noise in human cognition: Is it ubiquitous, and what does it mean?. Psychonomic Bulletin & Review.

[CR24] Gilden DL (2001). Cognitive emission of 1/*f* noise. Psychological Review.

[CR25] Gilden DL (2009). Global analysis of cognitive variability. Cognitive Science.

[CR26] Gilden DL, Thornton T, Mallon MW (1995). 1/*f* noise in human cognition. Science.

[CR27] Goldberger AL, Amaral LAN, Hausdorff JM, Ivanov PC, Peng C-K, Stanley HE (2002). Fractal dynamics in physiology: Alternations with disease and aging. Proceedings of the National Academy of Sciences.

[CR28] Hasson U, Landesman O, Knappmeyer B, Valines I, Rubin N, Heeger DJ (2008). Neurocinematics: The neuroscience of film. Projections: The Journal for Movies and Mind.

[CR29] Hasson U, Malach R, Heeger DJ (2009). Reliability of cortical activity during natural stimulation. Trends in Cognitive Sciences.

[CR30] Hausdorff JM (2007). Gait dynamics, fractals, and falls: Finding meaning in the stride-to-stride fluctuations of human walking. Human Movement Science.

[CR31] Henderson JM, Chanceaux M, Smith TJ (2009). The influence of clutter on real-world scene search: Evidence from search efficiency and eye movements. Journal of Vision.

[CR32] Hochberg, J., & Brooks, V. (1996). The perception of motion pictures. In E. C. Carterette & M. P. Friedman (Eds.), *Handbook of perception & cognition* (Cognitive ecology, Vol. 10, pp. 205–292). San Diego, CA: Academic Press.

[CR33] Hoop B, Burton MD, Kazemi H, Khoo MCK (1996). Fractal noise in breathing. Bioengineering approaches to pulmonary physiology and medicine.

[CR34] Howard CJ, Holcombe AO (2010). Unexpected changes in direction of motion attract attention. Attention, Perception, & Performance.

[CR35] Hurst HE, Black RP, Simaika YM (1965). Long-term storage: An experimental study.

[CR36] Karagiannis T, Faloutsos M, Riedl RH (2002). Long-range dependence: Now you see it now you don’t. IEEE Global Telecommunications Conference.

[CR37] Kaufman JC, Simonton DK (2014). The social science of cinema.

[CR38] Levin, D. T., & Baker, L. J. (2017). Bridging views in cinema: A review of the art and science of view integration. *WIREs Cognitive Science, 8*(5). 10.1002/wcs.1436 10.1002/wcs.1436 28263033

[CR39] Little MA, McSharry PE, Roberts SJ, Costello DAE, Moroz IM (2007). Exploiting nonlinear recurrence and fractal scaling properties for voice disorder detection. BioMedical Engineering OnLine.

[CR40] Magliano JP, Zacks JM (2011). The impact of continuity editing in narrative film on event segmentation. Cognitive Science.

[CR41] Mandelbrot B (1983). The fractal geometry of nature.

[CR42] Mandelbrot B (1999). Multifractals and 1/f noise: Wild self-affinity in physics.

[CR43] Marr D (1982). Vision.

[CR44] Mital PK, Smith TJ, Hill RL, Henderson JM (2011). Clustering of gaze during dynamic scene viewing is predicted by motion. Cognitive Computation.

[CR45] Münsterberg, H. (1915). Why we go to the movies. *Cosmopolitan, 60*(1), 22–31. Reprinted in Corrigan, T., White, P., & Mazaj, M. (Eds.) (2010). *Critical visions in film theory* (pp. 10–16). New York, NY: Bedford/St. Martin’s Press.

[CR46] Münsterberg H (1916). The photoplay: A psychological study.

[CR47] Newman MEJ (2005). Power laws, Pareto distributions, and Zipf’s law. Contemporary Physics.

[CR48] Nitzany EI, Victor JD (2014). The statistics of local motion signals in naturalistic movies. Journal of Vision.

[CR49] Parker, C. (2012). What makes: A director good or a good director? *Director Speak*. https://catlander.wordpress.com/2012/08/20/what-makes-a-director-good-or-a-good-director/. Accessed 11 Jan 2018.

[CR50] Pearlman K (2017). Editing and cognition beyond continuity. Projections: The Journal for Movies and Mind.

[CR51] Pearlman K (2009). Cutting rhythms: Shaping the film edit.

[CR52] Polking K (1990). Writing A to Z: The terms, procedures, and facts of the writing business defined, explained, and put within reach.

[CR53] Pressing, J., & Jolley-Rogers, G. (1997). Spectral properties of human cognition and skill. *Biological Cybernetics, 76*, 339–347. 10.1007/s004220050347. 10.1007/s004220050347 9237359

[CR54] Richmond LL, Gold DA, Zacks JM (2017). Improving event cognition: From the laboratory to the clinic. Journal of Applied Research in Memory and Cognition.

[CR55] Rosenholtz R, Li Y, Nakano L (2007). Measuring visual clutter. Journal of Vision.

[CR56] Salt B (2006). Moving into pictures.

[CR57] Salt B (2009). Film style and technology: History and analysis.

[CR58] Salt, B. (2010). *Comments on Attention and Hollywood films*. http://www.cinemetrics.lv/salt_on_cutting.php. Accessed 1 Aug 2017.

[CR59] Sargent, J. Q., Zacks, J. M., Hambrick, D. Z., Zacks, R. T., Kurby, C. A., Bailey, H. R., …Beck, T. M. (2013). Event segmentation ability uniquely predicts event memory. *Cognition, 129*(2), 241–255. 10.1016/j.cognition.2013.07.002. 10.1016/j.cognition.2013.07.002 PMC3821069 23942350

[CR60] Shimamura A (2013). Psychocinematics: Exploring cognition at the movies.

[CR61] Shimamura AP, Cohn-Sheehy BI, Pogue BL, Shimamura TA (2015). How attention is driven by film edits: A multimodal experience. Psychology of Aesthetics, Creativity, and the Arts.

[CR62] Shimotsu K, Phillips PCB (2005). Exact local Whittle estimation of fractional integration. The Annals of Statistics.

[CR63] Simonsohn, U. (2017). Two lines: The first valid test of U-shaped relationships. https:/ssrn.com.abtsract=3021690. Accessed 9 Oct 2017.

[CR64] Smith TJ (2012). The attentional theory of cinematic continuity. Projections: The Journal for Movies and Mind.

[CR65] Smith, T. J. (2013). Watching you watch movies: Using eye tracking to inform cognitive film theory. In A. P. Shimamura (Ed.), *Psychoncinematics: Exploring cognition at the movies* (pp. 165–191). New York, NY: Oxford University Press. 10.1093/acprof:oso/9780199862139.003.0009.

[CR66] Smith TJ, Henderson JM (2008). Edit blindness: The relationship between attention and global change blindness in dynamic scenes. Journal of Eye Movement Research.

[CR67] Smith TJ, Levin DT, Cutting JE (2012). A window on reality: Perceiving edited moving images. Current Directions in Psychological Science.

[CR68] Spehar B, Owens C (2013). When do luminance changes capture attention?. Attention, Perception & Psychophysics.

[CR69] Stadnitski T (2012). Measuring fractality. Frontiers of Physiology.

[CR70] Stadnitski T (2012). Some critical aspects of fractality research. Nonlinear Dynamics, Psychology, and Life Sciences.

[CR71] Tagliazucchi R, von Wegner F, Morzelewski A, Brodbeck V, Jahnke K, Laufs H (2013). Breakdown of long-range temporal dependence in default mode and attentional networks during deep sleep. Proceedings of the National Academy of Sciences.

[CR72] Theeuwes J (1991). Exogenous and endogenous control of attention: The effect of visual onsets and offsets. Attention, Perception & Psychophysics.

[CR73] Thompson K (1999). Storytelling in the new Hollywood.

[CR74] Thornton TL, Gilden DL (2005). Provenance of correlations in psychological data. Psychonomic Bulletin & Review.

[CR75] Van Orden, G. C. (2007). The fractal picture of health and wellbeing. *Psychological Science Agenda*, *21*(2). http://www.apa.org/science/about/psa/2007/02/van-ordern.aspx. Accessed 10 Jan 2018.

[CR76] von Mühlenen A, Lleras A (2007). No-onset looming motion guides spatial attention. Journal of Experimental Psychology: Human Perception and Performance.

[CR77] Wagenmakers E-J, Farrell S, Ratcliff R (2005). Human cognition and a pile of sand: A discussion on serial correlations and self-organized criticality. Journal of Experimental Psychology: General.

[CR78] West G (2017). Scale.

[CR79] Whittle P (1951). Hypothesis testing in time series analysis.

[CR80] Wijnants ML, Bosman AMT, Hasselman F, Cox RFA, Van Orden GC (2009). 1/*f* scaling in movement time changes with practice. Nonlinear Dynamics, Psychology, and Life Sciences.

[CR81] Zacks JM, Speer NK, Swallow KM, Maley CJ (2010). The brain’s cutting-room floor: Segmentation of narrative cinema. Frontiers in Human Neuroscience.

[CR82] Zacks JM, Swallow KM (2007). Event segmentation. Current Directions in Psychological Science.

[CR83] Allers R, Minkoff R (1994). The Lion King.

[CR84] Bradbury RN (1935). Westward Ho.

[CR85] Docter P, Del Carmen R (2015). Inside Out.

[CR86] Edwards B (1975). The Return of the Pink Panther.

[CR87] Howard R (1995). Apollo 13.

[CR88] Huston J (1950). The Asphalt Jungle.

[CR89] Kershner I (1980). Star Wars: Episode 5 – The Empire Strikes Back.

[CR90] Kostner K (1990). Dances with Wolves.

[CR91] Lester R (1980). Superman II.

[CR92] McCarey L (1945). The Bells of St. Mary’s.

[CR93] McQuarrie C (2015). Mission: Impossible – Rogue Nation.

[CR94] Wood S (1935). A Night at the Opera.

[CR95] Yates D (2010). Harry Potter and the Deathly Hallows: Part 1.

[CR96] Zemeckis R (1985). Back to the Future.

